# Selections

**Published:** 1816-01

**Authors:** 


### PART III.

### SELECTIONS.

### FRENCH DICTIONARY OF MEDICAL SCIENCES.

*Preliminary Remarks.*—The French Dictionary is a work to which few medical men, practising in these islands, can have access. The magnitude of the publication, and the labor and expence attendant on such an enterprize, oppose an insuperable bar to its translation into the English language. Animated by the desire of placing within the reach of his countrymen whatever of real value and novelty this diffuse work may contain, one of the Editors of this Journal has undertaken the task of presenting an analysis of its most instructive articles. Inordinate expectation invariably leads to disappointment. We wish, therefore, that the reader may clearly understand the principles upon which our analysis of this great work will be conducted. To have attempted a regular translation of the whole, even in the utmost possible state of condensation, would have been an enterprize boundless as extravagant. Intrinsic value of the articles will constitute the criterion by which our Selection will be determined: although no very long article will be passed over without some notice. We shall adopt the strictly analytical method; and endeavour to proportion the length of examination to the importance of the subject. Language the most simple, perspicuous, and concise will be employed. On some occasions, it may be necessary to present a nearly literal translation of the original: all such passages will be marked by inverted commas. In general, however, we may be regarded as condensing, in our own way and language, the opinions of the French writer, so as to present a rapid but faithful outline of the original. Our critical remarks will be few and brief; and these, in order to distinguish them from the substance of the article under discussion, will be placed in notes.

---

We commence with the Article Asphyxia,* compounded of α privative, and σφυξις, the pulse. "This word," says the writer, Savary, "then properly means cessation of the pulse, or suspension of the heart's action; and should be restricted to the state in which, consequently on this suspension, the functions of the other

---

* In general, our analysis will be pursued in the alphabetic order. On the present occasion, we are induced by circumstances, which we need not explain, to deviate from it: and this again may happen, whenever any subject treated in the dictionary, acquires, from events or controversies arising in the republic of medicine, an aspect of peculiar interest.

organs, essential to life, cease. But the term is applied to every species of apparent death; and, in latter times, a signification has been given to it, exactly the inverse of that which its etymology indicates. Thus, asphyxia is now defined to be the suspension of the phænomena of respiration; and consequent cessation of the cerebral, circulatory, and other functions."

"Syncope, like asphyxia and apoplexy, induces a state of apparent death. But in the first, according to Bichat, death begins by the heart; in the second, by the lungs; in the third, by the brain."

In this sense only, our author determines to employ the term asphyxia. The signs which distinguish it from real death, are elsewhere to be considered. It is here to be contemplated in a physiological and pathological view.—"Many physiologists, as Sauvages, Linnæus, Sagar, Vogel, and Macbride, placed asphyxia in the class of asthenic diseases. By Cullen, it was referred partly to apoplexy, partly to syncope; according as the death begins by the brain or heart. All authors have formed incorrect notions on the subject. Goodwin believed, that asphyxia consisted solely in the change of the arterial blood—that is, the presence of black blood in the left cavities of the heart; and proposed to call it *melanæma*, and refer it to the class cachexiæ. Pinel at first ranked it among comatose affections; but, in the later editions of his Nosography, he has correctly placed it with the diseases which essentially affect respiration. Several of the species of asphyxia really belong to the class of neuroses, where he has left them."

"Asphyxia then consists in the interruption or supension of the phænomena of respiration. These phænomena are of two kinds, mechanical and chemical. To the first, belong the movements of the thoracic parietes, called inspiration and expiration; to the latter, the absorption of oxygen gas,* extrication of carbonic acid, and conversion of the venous into arterial blood. Asphyxia may commence by the cessation of either of these phænomena: when it has taken place, both are equally suspended."

"A strong compression of the thoracic parietes; a wound exposing both cavities to the access of the air; rupture of the diaphragm with protrusion of the abdominal viscera into the chest, obstruct the movement of its parietes, the expansion and contraction of the lungs, and the action of the air upon the contained blood. In other cases, the motions of the chest terminate from paralysis of its muscles. This happens when the spinal marrow is divided or compressed above the third cervical vertebra; when the

---

* This is no longer the doctrine of Bristish physiologists. More recent experiments of our chemical philosophers have rendered it highly probable, if not absolutely proved, that no absorption of oxygen by the pulmonary blood takes place; and that the conversion of that fluid from the venous to the arterial character is principally operated by the extrication of carbon.

muscular system is struck by cold or a powerful electric shock; or is in a state of extreme debility, as happens in new-born children.

"Most frequently, however, the chemical phænomenon of respiration terminates first; and the motions of the thorax are interrupted, because the black blood, distributed to the muscles in common with the other organs, possesses not the excitement necessary to support their functions. This second order of causes presents numerous varieties. Thus, there may be absence of aeriform fluid in the lungs; as, when an animal is placed in vacuo, suffocated, drowned, or strangled. Again, the fluid inspired may be destitute of the vivifying principle which should act upon the blood contained in the capillary system of the lungs, and give it the arterial character; of this nature are nitrogen gas, hydrogen, nitrous oxide, carbonic acid, and the air spoiled by respiration or combustion. Lastly, the aeriform fluid, received into the lungs, may possess pernicious qualities, and produce irritation on the respiratory organs; as, the sulphureous acid, oxymuriatic acid,* and ammoniacal gases; or they may exert a deleterious influence on the whole economy: nitrous acid gas, carburetted hydrogen, sulphuretted hydrogen, hydro-sulphuret of ammonia, and, probably, arseniated hydrogen, operate in this manner. These considerations furnish a tolerably natural classification of the different species of Asphyxia. We present it in the form of Table.

I. Asphyxia from a mechanical Obstacle to Respiration.

1. From compression of the thorax and abdomen.—2. From admission of air into both cavities of the pleura.—3. From wound of the diaphragm, with escape of the abdominal viscera into the chest.

II. Asphyxia from Inaction of the Muscles of Inspiration.

1. From division of the spinal marrow.—2. From lightning.—3. From cold.—4. From general debility (asphyxia of new-born children.)

III. Asphyxia from Privation of Air.

1. From vacuum, or rarefaction of the air.—2. From suffocation.—3. From drowning.—4. From strangulation.

IV. Asphyxia from Defect of respirable Air.

1. From nitrogen gas.—2. From hydrogen gas.—3. From nitrous oxide.—4. From carbonic acid gas.—5. From air spoiled by respiration or combustion.

V. Asphyxia from irritating Gases.

1. From sulphureous acid gas.—2. From oxymuriatic acid gas, (chlorine).—2. From ammoniacal gas.

VI. Asphyxia from deleterious Gases.

1. From nitrous acid gas.—2. From carburetted hydrogen gas,

---

* The chlorine of Sir H. Davy.

and carbonous oxide. — 3. From sulphuretted hydrogen gas. — 4. From hydro-sulphuret of ammonia.— 5. From arseniated hydrogen gas.

Asphyxia from mechanical obstacles to respiration. Such cases are rare. The various ways in which asphyxia may be thus produced are noted in the preceding table. Bichat cites two examples of immediate death from rupture of the diaphragm. When, in consequence of such accident, an abdominal viscus may have escaped into the chest, replacement of it, by changing the posture of the patient, might possibly be accomplished; but assistance must be promptly administered. The symptoms of this kind of asphyxia have not been observed.

Asphyxia from inaction of the inspiratory muscles. 1. *From division of the spinal marrow.* When section of the spinal marrow is performed above the first dorsal vertebra, the motions of the ribs cease, and respiration is carried on by the diaphragm alone. If the section be made between the third and fourth cervical vertebræ, respiration is more impeded, and soon stops altogether. It is destroyed at once, if the spinal marrow be cut between the occiput and first vertebra. These phænomena are explained, by considering the origin and distribution of the intercostal and diaphragmatic nerves. They may arise accidentally from wounds of the cervical region, or luxations of the atlas. Art can do nothing. In these cases, the actions of the heart, and functions of the organs of sense seated in the head, survive, for some moments, the death of the lungs.—2. *Asphyxia from lightning.* Effects various. Life is sometimes instantly annihilated: sometimes the animal and organic functions are merely suspended. Remedies: Stimulants, electricity. Struve recommends inhuming the body up to the chin in fresh earth. Various irritants may, at the same time, be applied.—*Asphyxia from cold.* General numbness, drowsiness, insensibility, are the precursory signs. The vital principle may lie dormant, and yet be not extinct, for one or two days. Treatment: The body to be plunged in snow; rubbed with this substance, with linens moistened in cold water, then in lukewarm water: the frictions to be directed from the epigastrium towards the extremities. Warmth returning, and the rigidity of the muscles yielding, the patient may be laid in a cold bed. Frictions to be continued. When the natural heat and flexility are restored, not before, stimulants; as irritations of the nostrils with a feather, with vapours of vinegar or ammonia, and internal aromatics, may be used.—4. *Asphyxia of new-born children.* This affection it is necessary to distinguish from apoplexy, their treatment being quite different. Difficult labours, attended with hæmorrhage; delicate constitution of the fœtus, compression of the umbilical chord during delivery, principally produce it. The infant is pale and livid; flesh flaccid; limbs flexile and motionless; no respiration; no pulsation in the chord or region of the heart; but, unless putrefaction have taken place, the treatment for asphyxia should be rigorously adopted.

The funis should not be divided, especially if the placenta still adhere. Immersion in warm water, with the addition of wine, not spirits; removal of clots and collections of mucus from the mouth and nostrils; excitement of sneezing; inflation of air into the lungs by a tube passed into the larynx; judicious application of cold, of cupping-glasses; pinching of the skin, suction of the breast, should be persevered in, during more than an hour, with due modifications. M. Golfin restored an infant, after all other means had failed, by laying it on the side, and thus causing a great quantity of fluid (liquor amnii) to flow from the trachea and bronchiæ, whereby respiration had been obstructed.

Asphyxia from privation of air. The cause of this phænomenon has only in latter times been correctly appreciated. Suffocation, drowning, strangulation, take place on the same principle. In all of them respiration ceases from want of aliment.

1. *Asphyxia in vacuo.* When the vacuum is progressively formed, the animal placed in it is affected with restlessness, agitation, hurried respiration; falls; is convulsed; passes the fæces involuntarily; often suffers hæmorrhage, and dies, never at a later period than eight or ten minutes. Sometimes resuscitation is practicable. In a vacuum suddenly formed, death is prompt and certain; and it is remarkable that, in this case, the animal dies when the pressure of the atmosphere of the recipient can yet sustain a column of twelve inches of mercury; while, in a vacuum gradually made, life remains until the pressure is reduced to five inches. Persons ascending very high mountains, and aëronauts, may, from the rarefaction which prevails in the higher strata of our atmosphere, experience somewhat similar effects. Great heat may produce rarefaction in an unpleasant degree; but this is a complicated effect, in which the elevation of temperature bears the predominant part.

(To be continued.)

---

### *Case of Poisoning by Prussic Acid, which terminated fatally in a few Minutes.**

A strong, healthy man, aged 36, took, one afternoon at two o'clock, under circumstances of desperation, greater part of the fluid contained in a small sealed phial: the quantity might be about an ounce. It exhaled a strong smell of bitter almonds. He staggered but few steps afterwards, sank on his knees, and fell without a groan. From four to five minutes afterwards, the physician, sent for, found him stretched out, and exhibiting no trace of pulse or respiration. In a few minutes, a horribly-deep expiration took place, whereby the ribs were drawn nearly to the vertebræ, and the chest appeared to form a hollow externally. The hands and feet

---

* Translated from a paper by Hufeland, in his *Journal der Practischen Heilkunde.* The translation is not literal; but all the important facts are stated with fidelity.

were death-cold; the countenance sunk, and of a dirty pale hue; the eyes half open, glistening, clear, and full of life, but inirritable; the mouth naturally closed; the abdomen and thorax yet warm, and covered with a clammy sweat; the face and forehead cold and dry.

Within a minute and a half, two other deep sonorous respirations followed, with a convulsive motion of the thoracic muscles: yet did not the mouth or any of the other muscles display the slightest action. On conveying him to a vault, after four hours, a faint groan, probably produced by a discharge of air from the chest, was again heard. In the evening, he was found stretched out; the eyes half open, full, prominent, and as brilliant as in the wildest emotions of ardent youth. The countenance had not the same dirty grey hue as before; but was spread over with a softened paleness which formed with the glistening eyes a peculiar and striking contrast. In the latter, not the faintest trace of irritability remained.

On the following day the body was examined. The countenance was not at all distorted or convulsed: it had the aspect of peaceful slumber. Scarcely any spasmodic contraction of the muscles could be observed in other parts, even in the fingers. The eyes were yet half open and bright. The whole back and neck were rigid; the mouth unchanged. Numerous spots* were observed upon the back; the abdomen somewhat drawn in; the arch of the chest unusually small. The body exhaled a strong smell of bitter almonds. On dividing the scalp, a great quantity of dark blue blood directly trickled out, and continued to escape while the cranium was sawn through, to the amount of more than two pounds. After removal of the skull-cap, the *dura mater* was seen covered with thick black blood, and all its vessels, as though injected; and before the membrane could be removed, more than twelve ounces of blood had flowed out from between the hemispheres of the brain and the falciform process: it was thick, dark blue, and smelt so strongly of bitter almonds as painfully to affect the nose. The *pia mater* and vessels of the brain were gorged with blood. The brain, natural in colour and consistence, displayed, upon cutting it in layers, both in the cortical and medullary substance, a great number of puncta oozing blood. In the left ventricle was found about half a table-spoonful of reddish watery fluid. The *plexus choroides* was turgid with blood. All the vessels in the basis of the brain and on the *tentorium cerebelli* were gorged and covered with extravasated blood. The cerebellum was natural in hue and consistence to its termination in the spinal marrow.

*Abdomen.* The omentum was found in a natural condition: the intestines small, drawn together, nowhere distended with air, but universally red, and here and there highly inflamed. The liver was natural in appearance; and, on incision, full of black fluid blood. The spleen was deep brown, congested with black blood, but not enlarged. The bladder full of urine: the stomach two-thirds filled with food. This mass, in which none of the ingredi-

---

* Literally death-spots: *i. e.* such as are commonly observed on dead bodies.

ents could be distinguished, had a strong odour of bitter almonds. The stomach itself was violently inflamed, and displayed here and there gangrenous spots of considerable size; the villous coat separated on the slightest touch with the nail. All the other abdominal viscera were sound.

*Thorax.* The lungs were without adhesion, but did not collapse to their ordinary volume; they were more solid in substance, more dense and heavy than common; their surface was somewhat red, and sprinkled here and there with black puncta. Internally, and especially towards the inferior and posterior parts, they were remarkably loaded with dark blue fluid blood of oleaginous consistence; so that the posterior surface, to the depth of about a line, seemed completely inflamed, and looked like the substance of the liver. The pericardium contained a few drops of the serous fluid. The heart itself was neither inflamed nor altered in structure: the anterior (right) ventricle, and posterior (left) auricle were full of thick black fluid blood. The arteries were empty; the venous system loaded with blood.

From every cavity of the body, especially the head and abdomen, a smell of bitter almonds was exhaled, so penetrating as painfully to affect the olfactory organs of the bye-standers. All the blood was of a dark blue colour, not coagulated; of the consistence of oil.

The appearances displayed in this examination by the head, lungs, and intestines, are such as we commonly find consequent on the action of narcotic poisons or charcoal vapours. But the condition of the blood was particularly remarkable, for its colour was not merely black, but such that, examined sideways, it looked as though tinged with a large proportion of Prussian blue. The smell of bitter almonds was retained by it after removal. Chemical analysis of the remaining poison shewed it to consist of Prussic acid in combination with alcohol.* The quantity of fluid swallowed might contain about forty grains of the acid. What rendered the rapid and destructive operation of the poison in this case more extraordinary was, that it went on, notwithstanding the stomach was nearly filled with food.

The observations of Dr. Hufeland are brief and unimportant. He considers that Prussic acid operates, like the poison of the viper, directly upon the blood; and destroys life by instantaneously robbing that fluid of its vital properties, and thus paralysing the heart. It is impossible that this effect could take place so rapidly through the medium of the nervous system; since, even ligature of the cardiac nerves would not so soon annihilate the functions of the heart. He concludes by proposing, as a new and difficult problem in state-medicine, the following queries: How is poisoning by Prussic acid to be distinguished? How to be obviated, since bitter almonds, Prussian blue, and Prussiate of potass, are within the reach of every one?

---

* Literally, a highly concentrated spirituous Prussic acid (acidum zootícum.)

